# Effect of ammonia-oxidizing bacterial strain that survives drought stress on corn compensatory growth upon post-drought rewatering

**DOI:** 10.3389/fpls.2022.947476

**Published:** 2022-09-15

**Authors:** Xiao-Ling Wang, Ke Ma, Lin Qi, Yu-Hua Liu, Jiang Shi, Xue-Lin Li, Li-Xia Zhang, Wei Liu, Peng Song

**Affiliations:** ^1^College of Agronomy, Henan University of Science and Technology, Luoyang, China; ^2^Henan Agricultural Broadcasting and Television School, Zhengzhou, China

**Keywords:** ammonia-oxidizing bacterial strain, coexistence, corn, compensatory growth, cytokinin, rewatering upon drought stress

## Abstract

A pot experiment was performed under rain–shelter conditions to explore the effects of drought stress and post-drought rewatering on the abundance of an ammonia-oxidizing bacteria (AOB) strain in corn (*Zea mays* L.) rhizosphere soils and the relationship between the AOB strain and corn (*Zea mays* L.) compensatory growth after drought stress rewatering. Corn seedlings were used as test materials, and one AOB strain was isolated and screened from the soil. The experimental design included six treatments: (1) wet (WT), (2) wet with AOB strain inoculation during wetness (WI), (3) wet with AOB strain inoculation during rewatering (WR), (4) post-drought rewatering (DT), (5) post-drought rewatering with AOB strain inoculation during wetness (DI), and (6) post-drought rewatering with AOB strain inoculation during rewatering (DR). Wetness and drought stress were obtained by keeping the soil water content at 75–80% and 50–55% of the field capacities, respectively. The results showed that the isolated and screened AOB strain (S2_8_1) had 100% similarity to *Ensifer sesbaniae*. The inoculation of S2_8_1 during the wet period in the DI treatment caused it to colonize the rhizosphere soil. Drought stress decreased its abundance, but rewatering resulted in a great increase. The S2_8_1 in the DI treatment increased the total biomass, water use efficiencies, net photosynthetic rates, rhizosphere soil nitrification rates, leaf cytokinin concentrations, xylem sap cytokinin concentrations, copy number of S2_8_1 in rhizosphere soils, and organic carbon contents in rhizosphere soils by 23, 104, 35, 30, 18, 29, 104, and 23% on day 10 after rewatering compared with WT treatment. In the DI treatment, the increase in rhizosphere soil nitrification rates caused by S2_8_1 during wetness was closely related to the cytokinin delivery from roots to leaves and increased leaf cytokinin concentrations. The increase in leaf cytokinin concentrations improved rewatering corn growth, which caused compensatory growth and increased water use. Compensatory and over-compensatory growths occurred in DT and DR treatments, respectively. Therefore, the coexistence of the strain of AOB with corn in rhizosphere soil increased the corn compensatory growth by regulating soil nitrification and root-induced leaf cytokinin.

## Introduction

The lack of rainfall and irrigation water is a serious threat to crop production in Northern China (Yan et al., 2018; Liu et al., 2020). Crop water use should be improved to meet the challenge of water shortage. For this reason, water-saving agricultural technologies, namely, supplemental irrigation, regulated deficit irrigation, deficit irrigation, and rainwater harvesting irrigation have been widely used in the region (Ali et al., 2019; Zhang et al., 2019a,b; Liu et al., 2021; Shi et al., 2021). Essentially, these water-saving techniques were all based on growth inhibition under drought stress and growth acceleration during subsequent rewatering, namely compensatory growth upon post-drought rewatering. Plant under-compensation, compensation, and over-compensation growths refer to the compensatory biomasses during recovery growth below, equal to, and exceeding the lost biomass during environmental stress, respectively (Belsky, 1986; Grogan and Zamin, 2018). Thus, research efforts in crop post-drought rewatering compensatory growth should continuously focus on the development of water-saving strategies in agricultural production.

Crop post-drought rewatering compensatory growth is the fast growth caused by rewatering stimulation, and the root is the most direct organ that receives the stimulation; therefore, crop post-drought rewatering is very important for compensatory regrowth. Wang et al. (2016, 2018a) reported that leaf cytokinin induced by its synthesis in the roots promoted fast corn growth during post-drought rewatering, and nitrate (${\text{NO}}_{3}^{-}$) in the soil is the key factor that promoted the synthesis of cytokinin in corn roots. Wang et al. (2020a,b) observed that ${\text{NO}}_{3}^{-}$ released from the soil, which was caused by rhizosphere soil nitrification, directly induced corn root cytokinin synthesis and its delivery to the leaves, thereby increasing post-drought rewatering compensatory growth. Furthermore, soil ammonia-oxidizing bacteria (AOB) play a key role in the compensatory growth of crops. Wang et al. (2021) reported that a strain of the AOB *Acinetobacter pittii*, which was isolated and screened from the soil, promoted rhizosphere soil nitrification and the compensatory growth of corn. However, the reports on the relationship between soil AOB and crop are limited. The present study aimed to reveal the crop post-drought rewatering compensatory growth mechanism based on the coexistence of soil AOB with the crop.

The rhizosphere is an area where many bacteria colonize and play a role in host plant growth (Gagné-Bourque et al., 2016); it is a good place for soil AOB to coexist with crops. Crops during post-drought rewatering experience soil wetness, drought stress, and rewatering. The variable soil water environments of crops under post-drought rewatering growth influence the AOB abundance because drought stress restrains the bacterial number, but wetness can play a positive role in it (Monokrousos et al., 2020). However, several studies have reported the abundance of soil AOB in crop rhizosphere during post-drought rewatering. The study of the relationship between the abundance of rhizosphere soil AOB with crop post-drought compensatory growth will reveal its mechanism from a coexisting view.

In the present study, corn seedlings were selected as the test materials because their seedling growth is susceptible to drought stress and rewatering. Corn is the largest crop in China and the third largest crop in the world. Determining its compensatory mechanism during post-drought rewatering is beneficial for agricultural production and the development of water-saving strategies. We intended to reveal a rhizosphere soil AOB strain that would survive drought stress, show increased growth with rewatering, and improve corn compensatory growth during post-drought rewatering. To test this hypothesis, we isolated and screened one strain of AOB from the soil of the study site to test the effect of soil AOB on corn growth. Then, the isolated AOB strain was inoculated into the soil to detect its abundance by quantitative fluorescence polymerase chain reaction (PCR). Quantitative PCR is an accurate method for detecting bacterial numbers in soils (Bland et al., 2021). The effects of drought stress and post-drought rewatering on the abundance of the isolated AOB strain in rhizosphere soils and the relationship between the abundance of the isolated AOB strain and soil nitrification rates in rhizosphere soils, leaf cytokinin, and leaf photosynthesis were investigated to understand the compensatory growth mechanisms.

## Materials and methods

### Experimental design

#### Isolation and screening of soil AOB strain

##### AOB strain isolation from soil

In the present study, a liquid medium containing 0.5 g (NH_4_)_2_SO_4_, 0.75 g KH_2_PO_4_, 0.25 g NaH_2_PO_4_, 0.01 g MnSO_4_·4H_2_O, 0.03 g MgSO_4_·7H_2_O, and 5.0 g CaCO_3_ dissolved in 1 L of distilled water was obtained to isolate and screen soil AOB strain, and the solution pH was regulated to 7.2. The 1 L of the liquid medium was dissolved in 2.5 g of agar to form a solid medium. A mixture of 5 g of yeast extracts, 10 g of NaCl, and 10 g of tryptone was dissolved in 1 L of distilled water with pH being regulated to 7.0 and served as the LB medium.

Soil samples were collected from the corn rhizosphere soil, and they were added to the liquid medium to cultivate for 15–25 days at 28 °C in the dark. Then, the liquid medium was examined with Griess reagent to qualitatively test the presence of the AOB strain. Griess reagent is a qualitative test reagent for nitrite; nitrite may be used to verify whether ammonia has been oxidized. The liquid medium (0.5 ml) was dropped onto a white porcelain colorimetric plate, followed by the addition of 0.1 ml of Griess reagent for colorization. The occurrence of red, pink, or dark red colors meant the presence of nitrite ions and the AOB strain due to the oxidation of ammonia into nitrite. Then, we obtain a small portion of the liquid medium and added it to the new liquid medium for another cultivation using the same method. We repeated this process two times to obtain the liquid medium with pure AOB. Several of these pure AOB liquid media was applied on the surface of a solid medium to perform a 5–8-day cultivation at 28°C under dark conditions. When tip-size bacterial colonies were observed, we selected a single colony and applied it to the surface of another solid medium. The pure bacterial colony was obtained after repeating this process 5–10 times. Finally, one strain (S2_8_1) of bacteria was selected as the experimental strain.

The pure bacterial colonies were subsequently inoculated into the liquid medium solution, which ultimately served as the AOB culture in the present study. In addition, a portion of the AOB culture was inoculated into the LB medium to detect the heterotrophic growth of the bacterial strain. The characteristics of individual AOB were observed under an optical microscope, and the isolated AOB strains were examined through Gram staining.

##### Nitrification rate detection

The purified AOB culture (1 ml) and its control consisting of 1 ml of liquid medium without AOB were applied to 50 g soil samples to test AOB effects on soil nitrification. The soil samples had an organic carbon content of 24.3 g·kg^−1^ and total nitrogen content of 2.2 g·kg^−1^. Then, 20 g of each soil sample was cultivated for 7 days at 25°C under a moisture level equal to 60% field capacity. The differences in soil ${\text{NO}}_{3}^{-}$ content between the inoculated soil samples and those not inoculated were divided by 7 (for the 7 days) to obtain the soil nitrification rate per day.

##### Identification of AOB strains

A bacterial genome extraction kit (Bioer Technology Co., Ltd.) was used to extract the total bacterial DNA, and 16Sr DNA was amplified. The forward and reverse primers were 27F (5′-AGAGTTGATCCTGGCTCAG-3′) and 1492R (5′-TACCTTGTTACGACTT-3′), respectively. The 25 μL of PCR reaction system components included 2.5 μL of 10 × Buffer (with Mg^2+^), 2 μL of dNTP (2.5 mmol L^−1^), 0.4 μL of each forward and reverse primer (10 μmol L^−1^), 30 ng DNA template, and 0.75 U Ex Taq DNA polymerase and the rest being double-distilled water (ddH_2_O) to reach a certain volume. The reaction procedure was as follows: pre-degeneration at 94°C for 4 min, degeneration at 94°C for 1 min, renaturation at 55°C for 45 s, extension at 72°C for 2 min, 30 circulations, and extension at 72°C for 10 min. We analyzed the amplification products using 1.0% agarose gels. DNA Fragment Purification Kit was used to purify a 1.5 kb target fragment. The purified products were sequenced by TinyGene Bio-Tech (shanghai) Co., Ltd. The genes of close strains were obtained by the comparative analysis of the DNA sequencing results with GenBank. Phylogenetic trees were constructed using the neighbor-joining method in MEGA3.1.

#### Corn compensatory growth of drought stress rewatering

**Figure 1:**
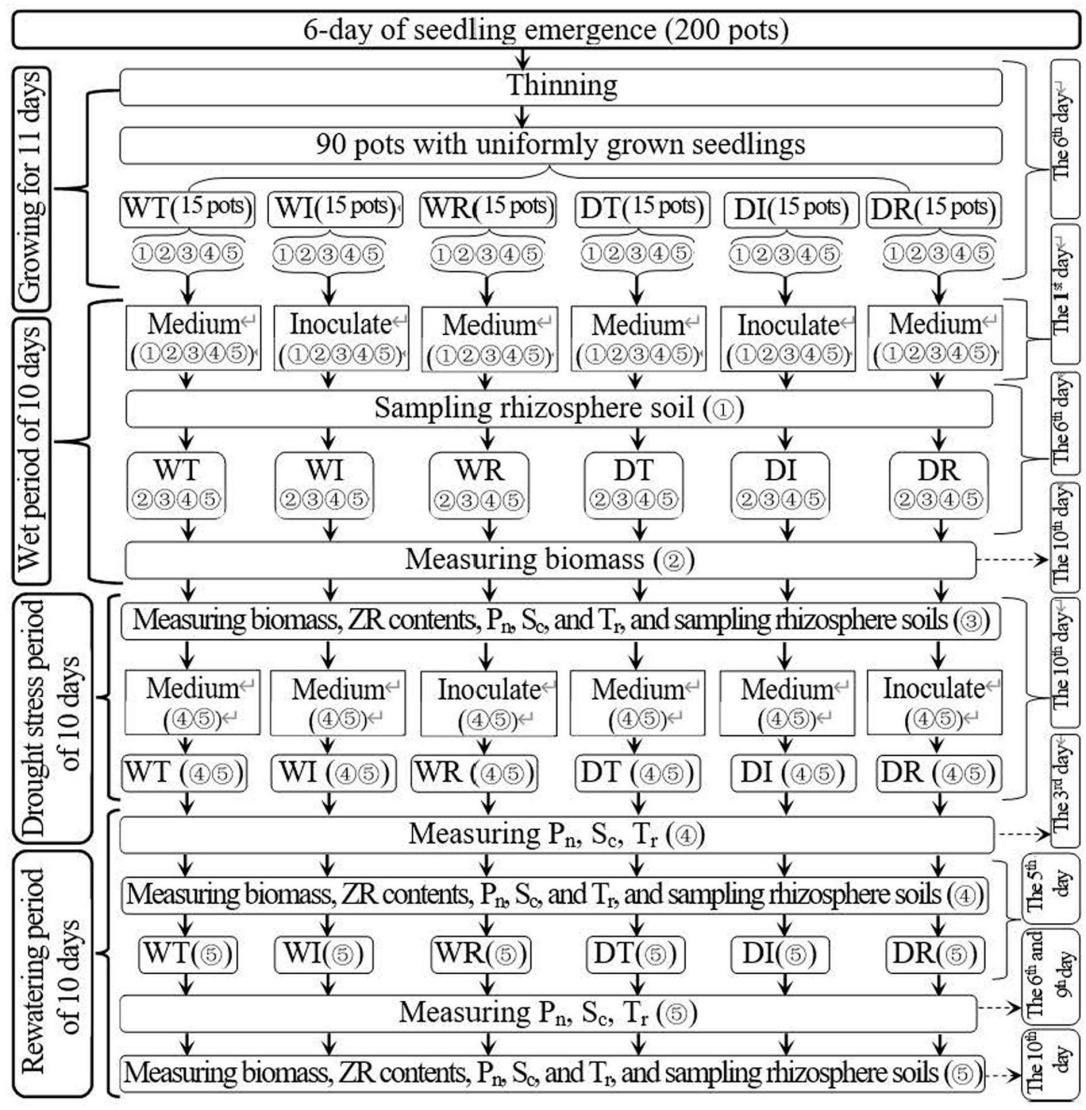
Schematic diagram for the experimental design. WT, WI, WR, DT, DI, and DR indicate treatments of wetness, wetness with AOB strain inoculation during wet period, wetness with AOB strain inoculation during rewatering period, post-drought rewatering, post-drought rewatering with AOB strain inoculation during wet period, post-drought rewatering with AOB strain inoculation during rewatering period, respectively. “①”, “②”, “③”, “④”, and “⑤” show the first, second, third, fourth, and fifth subgroups of each treatment, respectively. Pn, Sc, and Tr represent the net photosynthetic rate, stomatal conductance, and transpiration rate, respectively. “Inoculate” and “Medium” represent adding AOB stain solution and liquid medium without AOB, respectively.

A pot experiment was performed under rain–shelter conditions at the Henan University of Science and Technology, Luoyang City, Henan Province, China, and the results showed an average annual rainfall of 601 mm, an average annual temperature of 14.2°C, and average annual sunshine of 2,204.9 h. The corn (*Zea mays* L.) variety “Zhengdan 958” was used in the present study because of its drought resistance and good adaptability. Figure 1 displays the trial time course, treatment setting, and related indicators measured. On 5 June 2021, 200 plastic pots, which had a mouth diameter of 21.5 cm and a pot height of 20.0 cm, were planted with 15 corn seeds each. Each pot contained about 5.8 kg of soil with an organic carbon content of 24.7 g/kg and total nitrogen content of 2.15 g/kg. Corn seedling emergence occurred after about 6 days.

On the 6th day after emergence, thinning was performed in each pot to leave five seedlings that were growing well. In addition, 90 pots with well-growing seedlings were selected for the study. The 90 pots were evenly divided into four groups with 15 pots each, corresponding to the six experimental treatments in the present study: (1) wet (WT), (2) wet with AOB strain inoculation during wetness (WI), (3) wet with AOB strain inoculation during rewatering (WR), (4) post-drought rewatering (DT), (5) post-drought rewatering with AOB strain inoculation during wetness (DI), and (6) post-drought rewatering with AOB strain inoculation during rewatering (DR). The 15 pots in each treatment were divided into five subgroups with three pots each. The three pots in each subgroup were the three replicates for each treatment during each measurement.

The 10-day growth period of wetness was from the 11th to the 22nd day after emergence. On the first day of the wet period, 200 ml of purified AOB culture (216090 ${\text{cfu}\cdot \text{ml}}_{-}^{-1}$) was added to the soils of DI and WI treatments to inoculate AOB, and 200 ml of liquid medium without AOB was added to the soils of other treatments. On the 6th day of the wet period, the first subgroup in each treatment was picked out to sample the rhizosphere soils that were used for the measurements of AOB stain number. At the end of the wet period, the second subgroup of each treatment was selected to measure the corn biomass, which was used for the measurement of corn water use efficiency.

On the 22nd to 42nd day after emergence, the 10-day growth periods of drought stress and rewatering were established. During the 10-day drought stress period, the drought stress experiment was conducted in the third, fourth, and fifth subgroups in DT, DI, and DR treatments. In addition, the third, fourth, and fifth subgroups of WT, WI, and WR treatments remained wet. At the end of the drought period, corn biomass, zeatin riboside (ZR) content, net photosynthetic rate (P_n_), transpiration rate (T_r_), and stomatal conductance (S_c_) were measured in the third subgroup of each treatment, and rhizosphere soils that were used for the measurement of soil nitrification rate, soil ${\text{NH}}_{4}^{+}$, and ${\text{NO}}_{3}^{-}$ contents and AOB stain number were also sampled.

In the next 10 days of the rewatering period, all groups maintained wetness. At the beginning of the rewatering period, approximately 200 ml of purified AOB culture (216090 ${\text{cfu}\cdot \text{ml}}_{-}^{-1}$) was added to the soils in the fourth and fifth subgroups of DR and WR treatments, and 200 ml of liquid medium without AOB was added to the soils in the fourth and fifth subgroups of other treatment groups. At 5 and 10 days after rewatering, corn biomass and ZR content were measured in the fourth and fifth subgroups of each treatment group, and rhizosphere soils were sampled. At 3 days after rewatering, P_n_, T_r_, and S_c_ were measured in the fourth subgroup of each treatment. The same procedure was performed in the fifth subgroup of each treatment group on days 6 and 9 after rewatering.

Water was applied in pots with a soil water content below 50% of the field capacity to maintain 50–55% of the field capacity. Consequently, drought stress was obtained. Similarly, the soil water content was maintained at 75–80% of the field capacity by adding water. Then, wetness was obtained. In the study of Wang et al. (2018b), the soil water contents are calculated using Formula (1):


(1) $$\begin{array}{r} SWC=\frac{B_{t}-B_{d}-B_{e}-B_{p}}{B_{d}\;\times \;FWC}\times 100\% \end{array}$$

where SWC, B_t_, B_d_, B_e_, B_p_, and FWC are the soil water content, temporary whole pot weight, net dried soil weight, empty pot weight, estimated fresh weight of all plants, and field water capacity in each pot, respectively. B_p_ was determined on extra pots early.

### Measurements and data analysis

#### Biomass, photosynthesis, soil nitrification, and zeatin riboside

The roots were washed with water to remove soil. The roots, stems, and leaves were dried for 72 h at 65°C to obtain dry matter. The aboveground and total biomasses were the sums of dry matters of the stem and leaf, and root, stem, and leaf, respectively. Water used throughout the drought stress and rewatering periods was divided by the total biomass increase to calculate the water use efficiency. The difference in total biomass between the start of the drought stress period and the end of the rewatering period was used to calculate the total biomass increase. Water use was calculated by adding water throughout the drought stress and rewatering periods. LI-6400 photosynthesis equipment was used at 11:00 am to measure P_n_, T_r_, and G_s_.

Soil ${\text{NH}}_{4}^{+}$ and ${\text{NO}}_{3}^{-}$ contents were measured using the indophenol blue method and phenol disulfonic acid colorimetry, respectively (Lu, 1999). Soil samples that retained 60% water content of the field capacity were cultured for 7 days at 25°C to determine the soil net nitrification rate. The differences in soil ${\text{NO}}_{3}^{-}$ content before and after culturing were divided by 7 days to obtain the daily soil net nitrification rate. Soil organic carbon (SOC) contents in rhizosphere soils were determined by the potassium dichromate outside heating method (Lu, 1999).

The corns were clipped at the stem base. Then, 1.0 g absorbent cotton was used to cover the wounds to absorb the xylem sap for 12 h. The xylem sap volume was obtained by dividing the cotton weight increase by 1 g/cm^3^. The cotton was compacted, and the saps were squeezed out. The saps were collected for the measurement of the ZR concentration. Enzyme-linked immunosorbent assay was used for the measurement of the ZR content in the leaf and xylem saps (C_ZR_) in accordance with the method of Qin and Wang (2020). The ZR content in the xylem saps that were collected per hour was used to express the ZR delivery rate from the roots to the leaves (R_ZR_).

#### Quantitative real-time PCR and analysis

The total DNA of the soil genome was extracted from rhizosphere soil samples using the MO-BIO PowerSoil DNA Isolation Kit and used as a template for PCR amplification. The specific primers F (5′-ATGTACTGCGCTCAAATCCGA-3′), R (5′-ATGATGAAGGCAAAACCACGAT-3′), and probe P (5′-FAM-ACAACGCAGAAGTCGCACGGAAG-BHQ1-3′) of S2_8_1 were used for the PCR amplification of genomic DNA targeting the gene of S2_8_1, which was obtained by 16Sr DNA amplification in “2.1.1.3 Identification of AOB strains.” The 25 μL of reaction system contained the following: 12.5 μL of Premix Ex Taq (qPCR probe) (2 ×), 0.5 μL of forward primer F (10 μM), 0.5 μL of reverse primer R (10 μM), 0.5 μL of probe (10 μM), 5 μL of DNA template, and 6 μL of ddH_2_O. The reaction conditions were pre-denaturation at 95 °C for 30 s, denaturation at 95 °C for 10 s, annealing at 60 °C for 45 s, and recycling for 45 times. Each soil sample was tested three times. The extracted total DNA of the soil genome was amplified, and the Ct value of the sample obtained by quantitative fluorescence PCR was introduced into the standard curve equation to calculate the copy number of S2_8_1 (copies/g) in the rhizosphere soil of maize. The copy number was used to indicate the bacterial number.

The specific primers of S2_8_1 were amplified using PCR to construct the standard curve. The amplified products were purified, and the plasmids were constructed and transformed into *Escherichia coli* competent cells. The *E. coli* containing the target gene plasmid in the clone library was cultured in a shake flask at 37°C. The plasmid was extracted by Axygen Plasmid Miniprep Kit (Axygen) kit. The plasmid concentration was determined by Qubit 3.0 (Life Biotech), and the copy number of the plasmid was calculated. The gradient dilution results of the standard plasmid were as follows (5–7 points were generally diluted, and the points with good qPCR were selected as the standard curves): 5.86 × 10^5^, 5.86 × 10^4^, 5.86 × 10^3^, 5.86 × 10^2^, and 5.86 copies/μL. Five standard samples with different concentrations were subjected to quantitative fluorescence detection to establish a linear relationship between the Ct value and the concentration of S2_8_1 strain in corn rhizosphere soil. The abbreviations used in the text are summarized in Table 1. All values given in the figure are average values. The general linear model in SPSS 23 was used to conduct a one-way analysis of variance followed by Dunnett's test at the 0.05 probability level.

**Table 1:** Symbol definition.

**Symbol**	**Definition**	**Symbol**	**Definition**
AOB	Ammonia oxidizing bacteria	B_t_	Temporary whole pot weight
S2_8_1	The AOB strains	B_d_	Net dried soil weight
${\text{NO}}_{3}^{-}$	Soil nitrate nitrogen	B_e_	Empty pot weight
${\text{NH}}_{4}^{+}$	Soil ammonium nitrogen	B_p_	Estimated fresh weight of all plants
WT	Wetness	ZR	Zeatin riboside
WI	Wetness with AOB strain inoculation during wet period	R_ZR_	Delivery rate of ZR from roots to leaves
WR	Wetness with AOB strain inoculation during rewatering period	C_ZR_	ZR concentration in xylem sap
DT	Post-drought rewatering	P_n_	Photosynthetic rate
DI	Post-drought rewatering with AOB strain inoculation during wet period	S_c_	Stomatal conductance
DR	Post-drought rewatering with AOB strain inoculation during rewatering period	T_r_	Transpiration rate
SWC	Soil water content	SOC	Soil organic carbon
FWC	Field water capacity	PCR	Polymerase chain reaction

## Results

### Isolation and screening of soil AOB strain

**Figure 2:**
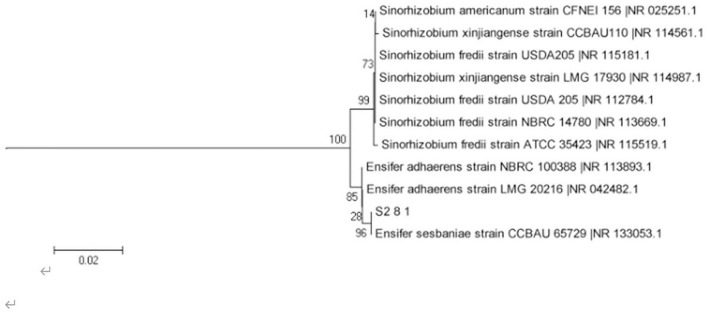
Phylogenetic tree based on the 16S rDNA sequence of 1 AOB strain. The S2_8_1 in the figure is the strain that has been isolated and screened in the present study.

**Figure 3:**
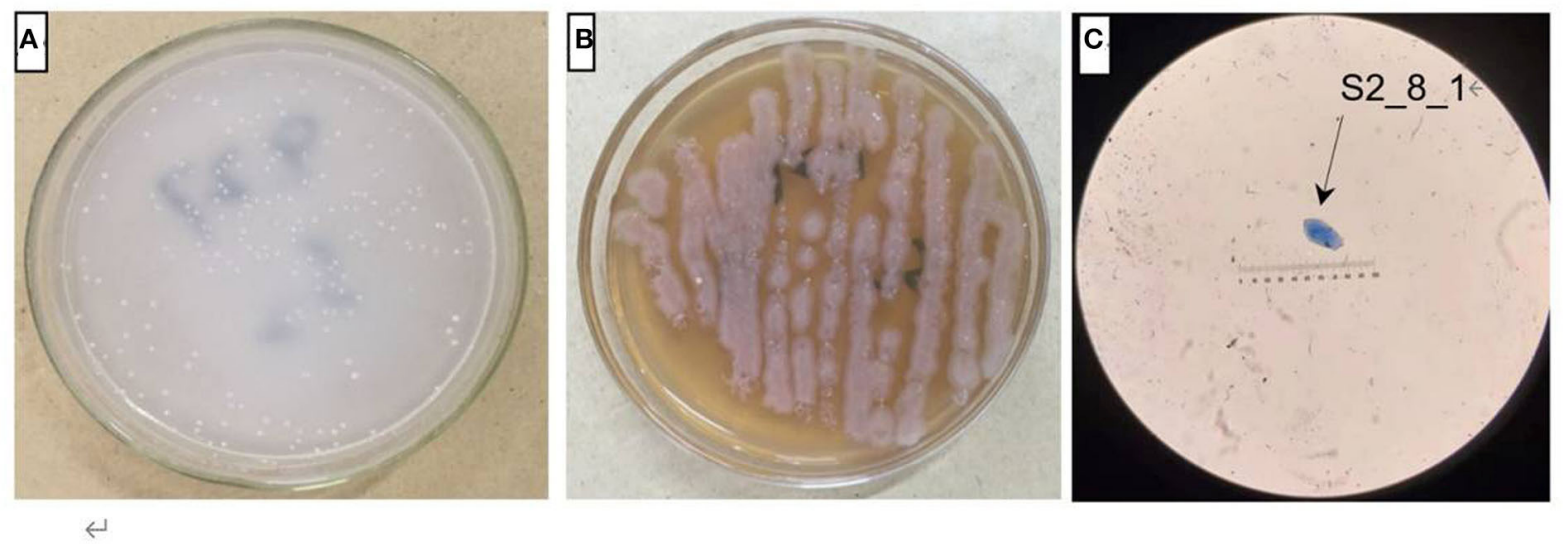
Cell and colonial morphology of strain S2_8_1. The S2_8_1 was the strain that has been isolated and screened in the present study. **(A,B)** showed the morphology characteristics of cell and colony of strain S2_8_1 in separation and LB mediums, respectively. **(C)** showed the size and sharp of strain S2_8_1, respectively.

The AOB strain S2_8_1 was isolated and screened in the present study. As shown in Figure 2, it belongs to *Ensifer* and has 100% similarity to *Ensifer sesbaniae*. Colonies appeared at 4–6 and 1–2 days after the inoculation of S2_8_1 into the separation and LB media, respectively (Figures 3A,B). The growth potential of S2_8_1 was higher in the LB medium than in the separation medium, which showed that the S2_8_1 had mixed nutritional characteristics and was inclined to be heterotrophic. On the AOB separation medium, the colonies of S2_8_1 appeared milky white and round with an irregular edge and smooth surface (Figure 3A). The S2_8_1 bacterium was 1.09-μm long and 0.54 μm wide and had a shape similar like a short bar under the microscope (Figure 3C). The soil nitrification rate was 10.89 and 5.89 mg/kg•d in soils with and without S2_8_1 added, respectively; and it was 1.85 times higher in soil with S2_8_1 added than that without S2_8_1 added. Therefore, S2_8_1 greatly increased the soil nitrification rate.

### Biomass

**Figure 4:**
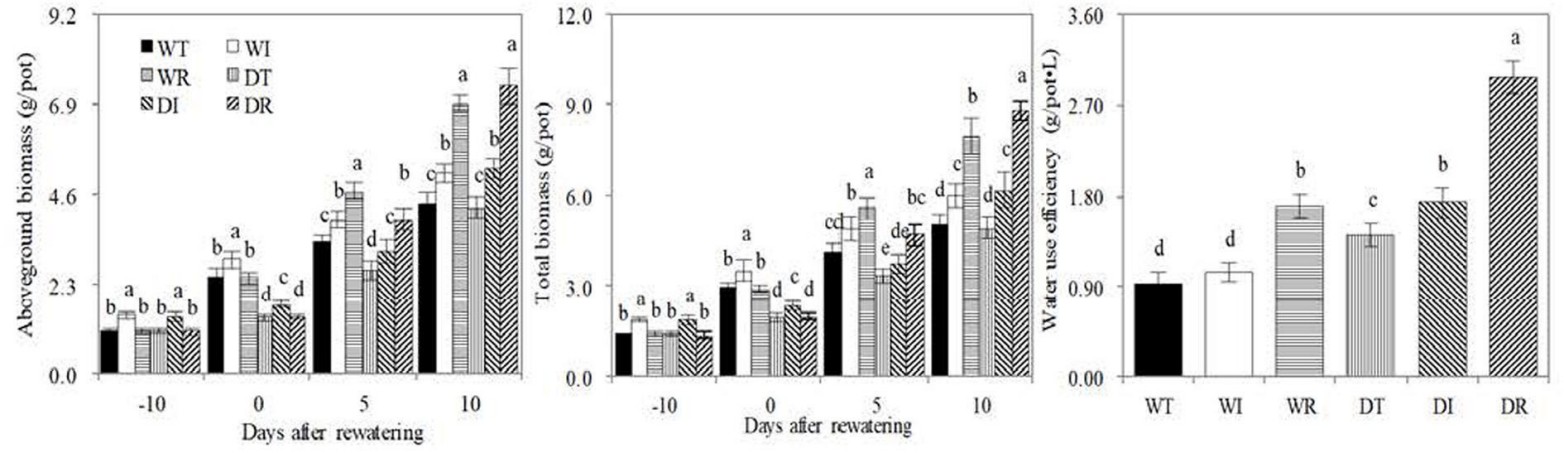
Biomass, water use efficiency in the different treatments in the study. WT, WI, WR, DT, DI, and DR indicate treatments of wetness, wetness with AOB strain inoculation during wet period, wetness with AOB strain inoculation during rewatering period, post-drought rewatering, post-drought rewatering with AOB strain inoculation during wet period, post-drought rewatering with AOB strain inoculation during rewatering period, respectively. In “−10” means the 10th day before the beginning of rewatering, namely the end of the wet period. In “0”, “5”, and “10” respectively stand for the beginning, 5th, 10th days of the 10-day rewatering period. The values are the mean ± standard error (*n* = 3). The different letters in each row indicate significant differences (*P* < 0.05).

The aboveground and total biomasses were significantly higher in WI and DI than in other treatments at the end of the wet period, and were significantly higher in WT than in DT, in WI than in DI, and in WR than in DR on day 0 after rewatering (Figure 4). These results showed that drought stress inhibited corn growth, whereas the inoculation of S2_8_1 during wet and drought stress periods increased corn growth. On the 10th day post-rewatering, similar aboveground and total biomasses were observed between WT and DT, and between WI and DI, and significantly higher total biomasses were observed in DR compared with WR. Therefore, rewatering increased corn growth. The total biomasses in DR on the 10th-day post-rewatering were 1.75, 1.79, and 1.43 times higher than those in WT, DT, and DI, respectively. Similarly, the total biomass in DI was 1.22 and 1.25 times higher than those in WT and DT, respectively. Thus, the inoculation of S2_8_1 during rewatering or wetness enhanced the growth of rewatering corn, but the inoculation during rewatering had a larger promotional effect than that during wetness.

The water use efficiencies in DR were 3.16, 2.82, 1.75, 2.09, and 1.72 times higher than those in WT, WI, WR, DT, and DI, respectively. The values were 1.84, 1.65, and 1.22 times higher in DI than those in WT, WI, and DT, respectively. In addition, water use efficiency was 1.51 times higher in DT than that in WT. Therefore, the inoculation of S2_8_1 during rewatering had the largest promotional effect on promoting water use, followed by the inoculation of S2_8_1 during wetness. Simple rewatering had the smallest effect.

### Photosynthetic characteristics

**Figure 5:**
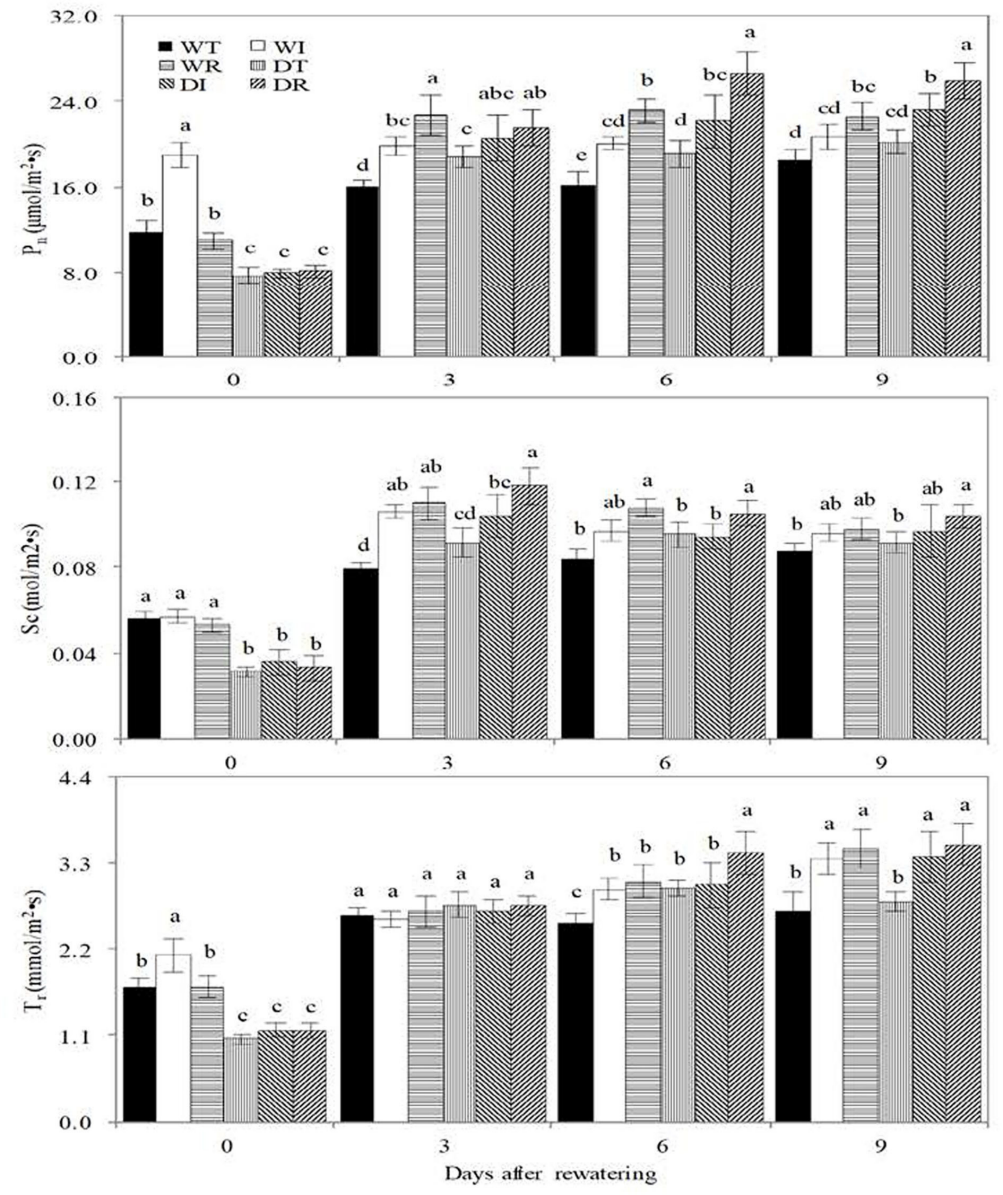
Biomass, water use efficiency in the different treatments in the study. WT, WI, WR, DT, DI, and DR indicate treatments of wetness, wetness with AOB strain inoculation during wet period, wetness with AOB strain inoculation during rewatering period, post-drought rewatering, post-drought rewatering with AOB strain inoculation during wet period, post-drought rewatering with AOB strain inoculation during rewatering period, respectively. In “0”, “3”, “6” and “9” respectively stand for the beginning, 3rd day, 6th day, 9th day of the 9-day rewatering period. Pn, Sc, and Tr represent the net photosynthetic rate, conductance, and transpiration rate, respectively. The values are the mean ± standard error (*n* = 3). The different letters in each row indicate significant differences (*P* < 0.05).

Before rewatering, significantly higher values of P_n_, T_r_, and S_c_ were observed in WT than in DT, in WI than in DI, and in WR than in DR, which showed that drought stress restrained the photosynthesis of corn (Figure 5). The value of P_n_ was significantly higher in DI and DR than in WT on the third, sixth, and 9th days after rewatering, in DT than in WT on the third and 6th days after rewatering, and in DI and DR than in DT on the 6 and 9th days after rewatering. S_c_ was significantly higher in DR than in DT and in WR than in WT on the third and 6th days after rewatering; it was significantly higher in DR than in DT and in WR than in WT on the third and 6th days after rewatering. T_r_ was significantly higher in DT than in WT on the 6th day after rewatering, in DI and DR than in DT on the 9th day after rewatering, and in WI and WR than in WT on the 6th and 9th days after rewatering. These results showed that rewatering and inoculation of S2_8_1 improved photosynthesis.

The inoculation of S2_8_1 during rewatering had a larger promotional effect on photosynthesis than that before drought stress because significantly higher P_n_ was observed in DR than in DI on the 6th and 9th days after rewatering, and significantly higher T_r_ and S_c_ were noticed in DR than in DI on the 6th day after rewatering.

### ZR, soil nitrification rate, soil ${\text{NO}}_{3}^{-}$, and ${\text{NH}}_{4}^{+}$ contents

**Figure 6:**
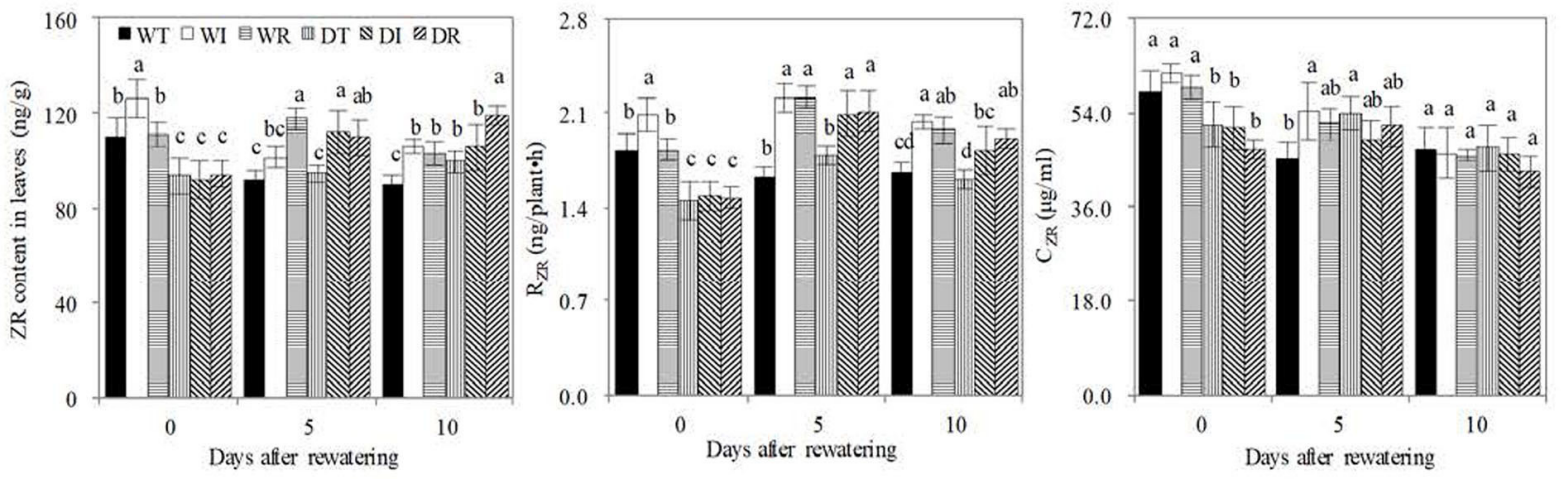
ZR, RZR, and CZR concentrations in the newly grown leaves. WT, WI, WR, DT, DI, and DR indicate treatments of wetness, wetness with AOB strain inoculation during wet period, wetness with AOB strain inoculation during rewatering period, post-drought rewatering, post-drought rewatering with AOB strain inoculation during wet period, post-drought rewatering with AOB strain inoculation during rewatering period, respectively. In “0”, “5”, and “10” respectively stand for the beginning, 5th day, 10th day of the 10-day rewatering period. CZR mean ZR concentrations in xylem sap. RZR mean delivery rates of ZR from roots to leaves, respectively. The values are the mean ± standard error (*n* = 3). The different letters in each row indicate significant differences (*P* < 0.05).

Before rewatering, the leaf ZR content, R_ZR_, and C_ZR_ of WT, WI, and WR significantly increased compared with those in DT, DI, and DR, respectively (Figure 6). Thus, drought stress decreased leaf cytokinin contents and delivery rates from the roots to the leaves. ZR is one of the major cytokinin forms. On the contrary, rewatering reversed this trend, with significant increases in the leaf ZR content found in DI compared with WI on the 5th day after rewatering and in DT and DR compared with WT and WR on the 10th day after rewatering, respectively. Non-significant lower R_ZR_ and C_ZR_ values were found in DT, DI, and DR than in WT, WI, and WR, respectively. In addition, the leaf ZR content and R_ZR_ were significantly higher in DR and DI than in WT and DT on the 5th or 10th days, which showed that S2_8_1 caused an increase in these indices of rewatering corn. The leaf ZR content was significantly higher in DR than in DI on the 10th day after rewatering. Therefore, the inoculation of S2_8_1 during rewatering had a larger promotional effect on leaf ZR content compared with inoculation during wetness.

**Figure 7:**
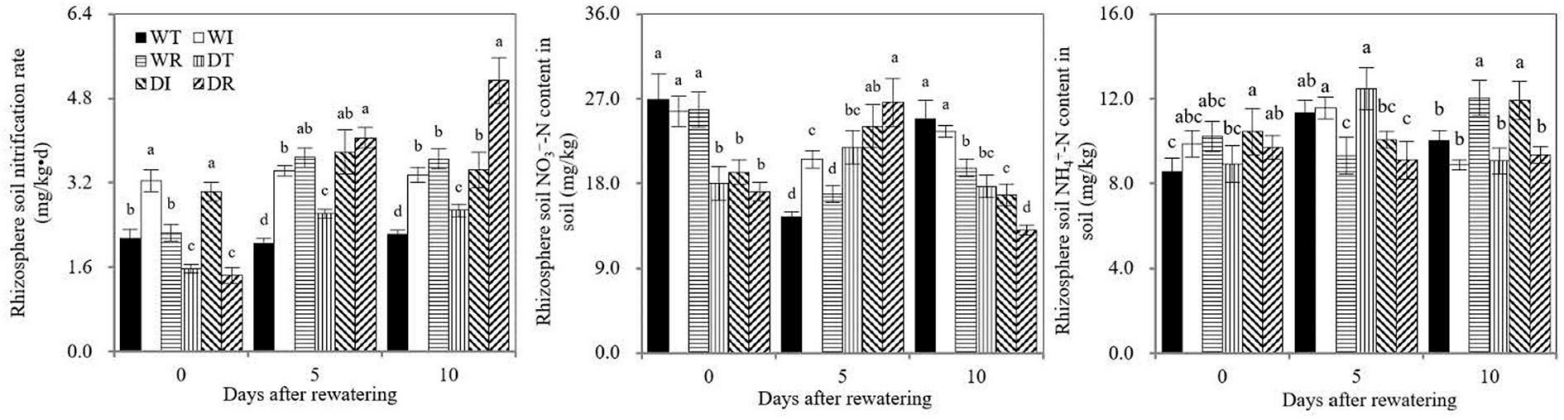
Soil nitrification rate, soil ammonium and nitrate nitrogen contents in the study. WT, WI, WR, DT, DI, and DR indicate treatments of wetness, wetness with AOB strain inoculation during wet period, wetness with AOB strain inoculation during rewatering period, post-drought rewatering, post-drought rewatering with AOB strain inoculation during wet period, post-drought rewatering with AOB strain inoculation during rewatering period, respectively. In “0”, “5”, and “10” respectively stand for the beginning, 5th day, 10th day of the rewatering period of 10 days. The values are the mean ± standard error (*n* = 3). The different letters in each row indicate significant differences (*P* < 0.05).

As shown in Figure 7, significant increases in rhizosphere soil nitrification rates were observed in the WT compared with DT and in WR compared with DR before rewatering, which indicated that drought stress inhibited rhizosphere soil nitrification. On days 5 and 10 after rewatering, significantly higher rhizosphere soil nitrification rates were found in DT than in WT, in DR and DI than in DT, and in WI and WR than in WT. Therefore, rewatering and S2_8_1 inoculation promoted rhizosphere soil nitrification. Significantly higher soil nitrification rates in the rhizosphere environments were detected in DR than in DI on the 10th after rewatering, which indicated that the larger promotional effect on the nitrification rate occurred when inoculating during rewatering than during wetness. During the rewatering period, irregular results, that is, by chance, were observed in rhizosphere soil ${\text{NO}}_{3}^{-}$ and ${\text{NH}}_{4}^{+}$ contents. Thus, the inoculation of S2_8_1 could not increase their contents.

### Strain number and SOC content in rhizosphere soil

**Figure 8:**
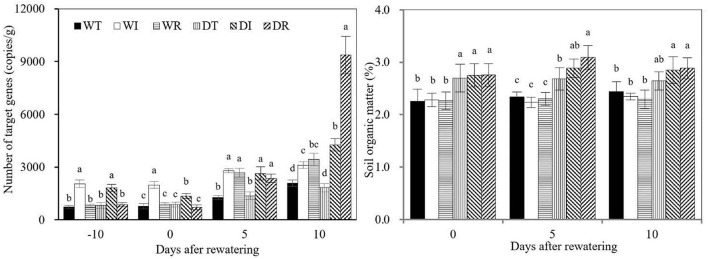
S2_8_1 number in rhizosphere soil and soil organic matter in the study. WT, WI, WR, DT, DI, and DR indicate treatments of wetness, wetness with AOB strain inoculation during wet period, wetness with AOB strain inoculation during rewatering period, post-drought rewatering, post-drought rewatering with AOB strain inoculation during wet period, post-drought rewatering with AOB strain inoculation during rewatering period, respectively. In “−10” means the 10th day before the beginning of rewatering, namely the end of the wet period. In “0”, “5”, and “10” respectively stand for the beginning, 5th day, 10th day of the rewatering period of 10 days. The values are the mean ± standard error (*n* = 3). The different letters in each row indicate significant differences (*P* < 0.05).

The copy number of S2_8_1 in rhizosphere soil was significantly higher in DI and WI than in WT, WR, DT, and DR during the wet period (Figure 8). Similarly, the copy number of S2_8_1 in DR and WR increased significantly compared with those in WT and DT during the rewatering period. Therefore, the inoculation of S2_8_1 increased its number in rhizosphere soil. The copy number of S2_8_1 was significantly higher in DR than in DI on day 10 after rewatering, which showed that inoculation during rewatering was more apt to increase the S2_8_1 amount in rhizosphere soil than inoculation during wetness. The copy number in WI was similar to that in DI during the wet period but was 1.5 times higher than that in DI at the end of the drought period. Thus, drought stress decreased the abundance of S2_8_1 in the rhizosphere soil. On the contrary, copy numbers of DI and DR were 1.4 and 2.7 times higher than that of WI and WR on day 10 after rewatering, respectively, which showed that rewatering increased the abundance of S2_8_1 in the rhizosphere soil.

Rhizosphere SOC contents were significantly higher in DT, DI, and DR than in WT, in DI than in WI, and in DR than in WR on days 0 and 5 after rewatering. Therefore, drought stress increased the rhizosphere SOC contents, and their lag effect continued until the rewatering period. On day 10 after rewatering, the rhizosphere SOC contents were significantly higher in DI and DR than in WT, WI, and WR, but similar SOC contents were observed between DT and WT. These results showed that rewatering corn used rhizosphere SOC. However, rewatering corn with S2_8_1 inoculation relatively maintained SOC stability.

## Discussion

### Coexistence of S2_8_1 and corn

In the present study, a stable coexisting relationship was found between corn and S2_8_1 in DI. Based on the copy number of S2_8_1 in rhizosphere soil, the inoculation of S2_8_1 in DI during wetness caused some of it to colonize in rhizosphere soil. Then drought stress decreased the abundance of S2_8_1 in rhizosphere soil, but rewatering greatly increased its abundance. In addition, S2_8_1 increased corn growth during the continued growth periods of wetness, drought stress, and rewatering.

Soil AOB comprise autotrophic, heterotrophic, and mixotrophic AOB (Taylor et al., 2009; Kouki et al., 2011; Matsuno et al., 2013). The present study isolated and screened the AOB strain S2_8_1, which belongs to *Ensifer* and has mixotrophic characteristics, but is inclined to be heterotrophic. Preece et al. (2018) and Gargallo-Garriga et al. (2018) reported that growing heterotrophic AOB use organic substances as the substrate. Carbon is the chief element of organic compounds, and SOC may represent the amount of organic compounds in the soil (Zhao et al., 2021). High rhizosphere SOC in DI and DR caused the increase in S2_8_1 abundance compared with WI and WR during the rewatering period, respectively. On day 10 after rewatering, more S2_8_1 were observed in DR compared with those in DI. The explanation for this was that given its S2_8_1 content being 216090 cfu·ml^−1^, the 200 ml of inoculation solution had a large number of S2_8_1. Therefore, under the condition of considerable rhizosphere organic substances caused by drought stress, more S2_8_1 easily colonized the rhizosphere micro-environment in DR compared with DI during the rewatering period. This finding further shows that rhizosphere soil organic matter was the key factor promoting S2_8_1 to colonize in the rhizosphere micro-environment.

Carbon compounds, namely, carbohydrates, organic acids, and alcohol, are secreted by plant roots into the rhizosphere soil under drought stress (Karlowsky et al., 2018; Calvo et al., 2019; De Vries et al., 2020). In the present study, the high SOC content in rhizosphere soil in DI, DR, and DT during the rewatering period was mainly because drought stress stimulated the release of a number of carbon compounds into rhizosphere soil and exerted a lag effect. As a result, rewatering increased the abundance of S2_8_1 in the rhizosphere soil in DI and DR. On the contrary, during the wet period, the relatively low rhizosphere SOC caused less colonization by the S2_8_1 strains compared with the rewatering period. Because the copy number of S2_8_1 of rhizosphere soil in DI was 2.3 times higher at the end of the rewatering period than that at the end of the wet period. During the drought stress period, although a high rhizosphere SOC was observed in the treatments of DI, the lack of water decreased the abundance of S2_8_1 compared with the rewatering period.

The S2_8_1 in the DI and DR during the rewatering period increased corn growth because on day 10 after rewatering, the total biomass of DI equaled that of WI, and it increased by 11% than in DR than in WR. As a result, compensatory and over-compensatory growths occurred in DI and DR, respectively. Compensatory and over-compensatory growths in DI and DR caused water use to increase greatly, as shown by the water use efficiencies which were 2.04 and 3.16 times higher in DI and DR than in WT, respectively, throughout the drought stress and rewatering periods. The high number of S2_8_1 in the rhizosphere soil increased the total biomass and water use efficiency in the DR by 1.43 and 1.56 times compared with those in DI on day 10 after rewatering, respectively, which further demonstrated the role of S2_8_1 in corn rewatering growth and water use. High total biomass in DI compared with DT during wet and drought stress periods showed that the presence of S2_8_1 increased corn growth in these periods.

### Root–shoot signaling of cytokinins

The present results revealed that the coexistence of S2_8_1 with corn in DI continuously increased the amount of root cytokinin delivered to leaves, thus improving corn rewatering growth and water use.

S2_8_1 caused the high rhizosphere soil nitrification rates in DI compared with DT or WT during rewatering because AOB are the main bacteria involved in soil nitrification. High rhizosphere soil nitrification rate caused the continuous release of more ${\text{NO}}_{3}^{-}$ from the soil during the rewatering period, increasing the stimulation by ${\text{NO}}_{3}^{-}$ in the roots. Soil ${\text{NO}}_{3}^{-}$ can stimulate roots to synthesize cytokinins in various plants (Landrein et al., 2018; Poitout et al., 2018). As a result, more root cytokinins are synthesized and continuously delivered to increase the leaf cytokinin content in the DI. Cytokinins synthesized in the roots can be transmitted to the leaves by means of the xylem sap (Zaicovski et al., 2008; Wang et al., 2016). *R*_ZR_ indicates the delivery speed of cytokinins from roots to leaves in the darkness. The high R_ZR_ in DI compared with DT and WT during the rewatering period demonstrated the additional cytokinins being synthesized and delivered to the leaves in darkness. The cytokinin delivery rate to the leaves in the presence of sunlight was the product of *T*_r_ and *C*_ZR_, and transpiration drove the sap to transmit from roots to leaves under this condition. More cytokinins were synthesized and delivered to the leaves in DI compared with DT and WT in sunlight during the rewatering period. Similar C_ZR_ but high T_r_ were present in DI compared with DT and WT during the rewatering period.

Similarly, the higher number of S2_8_1 in DI compared with WI and in DR compared with WR, WT, and DT all caused the high rhizosphere soil nitrification rate, increased synthesis of root cytokinin and delivery to leaves, and rapid corn growth during the rewatering period. Wang et al. (2020a) reported a higher rhizosphere soil nitrification rate in corns after post-drought rewatering compared with wet corns. It was likewise that rewatering increased the rhizosphere soil nitrification rates of DT compared with WT in the present study. This condition caused more cytokinins to be delivered in DT compared with WT during the rewatering period, increasing the leaf cytokinin content and corn growth. Thus, compensatory growth and high water use efficiency occurred in DT compared with WT.

The similar copy numbers of S2_8_1 in WT and DT contributed less to the result of high rhizosphere soil nitrification rate in DT compared with WT during the rewatering period. Because no inoculation caused the low number of S2_8_1 in the rhizosphere soil of WT and DT; thus, it was hard to detect accurately. The potential role of heterotrophic AOB on the rhizosphere soil of post-drought rewatering crops required further investigation in the future.

The high leaf cytokinin content in DI during rewatering was beneficial for the increase in leaf P_n_ compared with DT, WI, and WT. Cytokinin plays a promotional role in photosynthesis (Kobayashi et al., 2012; De Moura et al., 2017). The occurrence of high P_n_ in DI compared with DT during rewatering led to compensatory growth as corn growth after rewatering was mainly involved in the fast photosynthetic organic matter accumulation. Similarly, given the high leaf P_n_ caused by cytokinin, compensatory and over-compensatory growths occurred in DT and DR, respectively.

In the study of Takei et al. (2001) on maize seedlings, cytokinins played essential roles in the plant response to nitrate, where they acted as secondary messengers in plants. Wang et al. (2020b) observed that root-originated cytokinins induced by the continued addition of ${\text{NO}}_{3}^{-}$ caused the compensatory growth of post-drought rewatering due to its short duration in soil. The continuous delivery of cytokinins from roots to leaves is significant in corn compensatory growth, and the repeated addition of ${\text{NO}}_{3}^{-}$ is an effective method to achieve this goal. However, the present study revealed that the coexistence of S2_8_1 with corn increased its compensatory growth in post-drought rewatering by inducing root cytokinin delivery to the leaves continuously.

The low increase effect of S2_8_1 on corn growth was observed in DI compared with DR. However, S2_8_1 coexistence with corn in DI showed significance for the feasibility of the technique when using soil heterotrophic AOB strains, which were isolated from soil similar to the present study in crop water use. Rainwater is the water source of crops in dry farming; most rainfall entering into soils is a process of post-drought rewatering (Barker, 2017; Preethi et al., 2020). However, the uncertainty of rainfall causes the difficult inoculation of soil heterotrophic AOB strains with crops of drying farms before raining. Similar to S2_8_1, the inoculation of soil heterotrophic AOB after emergence would cause coexistence with the crop, which would increase rainfall use in a timely manner. Similarly, most crop irrigation is also a process of post-drought rewatering. The irrigation is usually accompanied by nitrogen fertilizer application to increase the crop water use efficiency (Tao et al., 2021; Zain et al., 2021). The coexistence of soil heterotrophic AOB with crops can increase the timely irrigation water use, which plays a potential role in replacing nitrogen fertilizer. For the application of our findings in the present study, field experiments should further be carried out based on our experimental results.

## Conclusion

The inoculation of S2_8_1 during wetness caused it to colonize the rhizosphere soil. Drought stress decreased its abundance, but rewatering greatly increased its abundance in rhizosphere soil. The S2_8_1 in rhizosphere soil during rewatering increased the soil nitrification rates, which played an important role in cytokinin synthesis in the roots and its delivery to the leaves. As a result, the cytokinin concentration in the leaves increased. The increase in corn leaf cytokinin caused by rewatering increased the photosynthetic rate, resulting in over-compensatory growth after post-drought rewatering and high water use efficiency. Thus, the coexistence of AOB strains with corn in rhizosphere soil increased corn compensatory growth by regulating soil nitrification and root-induced leaf cytokinin. Our findings are beneficial for developing strategies to increase crop water use.

## Data availability statement

The original contributions presented in the study are publicly available. This data can be found here: NCBI, ON667919.

## Author contributions

X-LW devised the project and supervised it. KM, Y-HL, LQ, and L-XZ conducted the experiments. PS, JS, and X-LL performed the isolation and screening of soil ammonia-oxidizing bacteria strains. WL analyzed the data. KM and LQ prepared the figures and wrote the manuscript.

## Funding

This work was funded by the National Natural Science Foundation of China (U1304326) and the Excellent Youth Foundation of Henan Scientific Committee (174100510004).

## Conflict of interest

The authors declare that the research was conducted in the absence of any commercial or financial relationships that could be construed as a potential conflict of interest.

## Publisher's note

All claims expressed in this article are solely those of the authors and do not necessarily represent those of their affiliated organizations, or those of the publisher, the editors and the reviewers. Any product that may be evaluated in this article, or claim that may be made by its manufacturer, is not guaranteed or endorsed by the publisher.
